# 基于深度学习的保留时间预测方法的研究进展及应用

**DOI:** 10.3724/SP.J.1123.2020.08015

**Published:** 2021-03-08

**Authors:** Zhuokun DU, Wei SHAO, Weijie QIN

**Affiliations:** 1.安徽医科大学基础医学院, 安徽 合肥 230032; 1. School of Basic Medicine, Anhui Medical University, Hefei 230032, China; 2.军事科学院军事医学研究院生命组学研究所, 北京蛋白质组研究中心, 蛋白质组学国家重点实验室, 北京 102206; 2. State Key Laboratory of Proteomics, Beijing Proteome Research Center, Beijing Institute of Lifeomics, Beijing 102206, China

**Keywords:** 液相色谱-串联质谱, 保留时间, 深度学习, 蛋白质组, liquid chromatography-tandem mass spectrometry(LC-MS/MS), retention time, deep learning, proteomics

## Abstract

在基于液相色谱-质谱联用的蛋白质组学研究中,肽段的保留时间作为有效区分不同肽段的特征参数,可以根据肽段自身的序列等信息对其进行预测。使用预测得到的保留时间辅助质谱数据鉴定肽段序列可以提高鉴定的准确性,因此对保留时间预测的工作一直受到领域内的广泛关注。传统的保留时间预测方法通常是根据氨基酸序列计算肽段的理化性质,进而计算肽段在特定色谱条件下的保留时间。近年来,深度学习方法取得了极大的进展,在蛋白质组学研究中发挥着越来越重要的作用。目前已发展出了多种基于深度学习的保留时间预测方法,与传统的保留时间预测方法相比有着更高的准确度,易于跨平台使用,并且能对修饰肽段的保留时间进行预测。但对某些复杂的修饰,如糖基化修饰等的预测结果还不够准确。如何进一步提高对修饰肽段预测的准确性是基于深度学习的保留时间预测方法的重要研究方向。这些预测的保留时间被应用于肽段鉴定的质量控制和方法评估,以及与预测的二级质谱谱图结合,建立模拟谱图库等方面。该文综述了深度学习方法在保留时间预测领域的最新研究进展以及应用成果,同时对其发展趋势和未来的应用方向进行了展望,以期为保留时间预测研究以及蛋白质组鉴定工作提供参考。

## Abstract (en)

In “shotgun” proteomics strategy, the proteome is explained by analyzing tryptic digested peptides using liquid chromatography-mass spectrometry. In this strategy, the retention time of peptides in liquid chromatography separation can be predicted based on the peptide sequence. This is a useful feature for peptide identification. Therefore, the prediction of the retention time has attracted much research attention. Traditional methods calculate the physical and chemical properties of the peptides based on their amino acid sequence to obtain the retention time under certain chromatography conditions; however, these methods cannot be directly adopted for other chromatography conditions, nor can they be used across laboratories or instrument platforms. To solve this problem, in recent years, deep learning was introduced to proteomics research for retention time prediction. Deep learning is an advanced machine-learning method that has extraordinary capability to learn complex relationships from large-scale data. By stacking multiple hidden neural networks, deep learning can ingest raw data without manually designed features. Transfer learning is an important method in deep learning. It improves the learning process a new task through the transfer of knowledge from an already-learned related task. Transfer learning allows models trained using large datasets to be utilized across conditions by fine-tuning on smaller datasets, instead of retraining the whole model. Many retention time prediction methods have been developed. In the process of training the model, the sequences of peptides are encoded to represent peptide information. Deep learning considers the relationship between the characteristics of the peptides and their corresponding retention times without the need for manual input of the physical and chemical properties of the peptides. Compared with traditional methods, deep learning methods have higher accuracy and can be easily used under different chromatography conditions by transfer learning. If there are not enough datasets to train a new model, a trained model from other datasets can be used as a replacement after calibration with small datasets obtained from these chromatography conditions. While the retention times of modified peptides can also be predicted, the predictions are inadequate for complex modifications such as glycosylation, and this is one of the main problems to be solved. The predicted retention times were used to control the quality of peptide identification. With high accuracy, the predicted retention times can be considered as actual retention times. Therefore, the difference between predicted and observed retention times can serve as an effective and unbiased quantitative metric for evaluating the quality of peptide-spectrum matches (PSMs) reported using different peptide identification methods. Combined with fragment ion intensity prediction, retention time prediction is used to generate spectral libraries for data-independent acquisition (DIA)-based mass spectrometry analysis. Generally, DIA methods identify peptides using specific spectrum libraries obtained from data-dependent acquisition (DDA) experiments. As a result, only peptides detected in the DDA experiments can be present in the libraries and detected in DIA. Furthermore, it takes a lot of time and effort to build libraries from DDA experiments, and typically, they cannot be adopted across different laboratories or instrument platforms. In contrast, the pseudo spectral libraries generated by retention times and fragment ion intensity prediction can overcome these shortcomings. The pseudo spectral libraries generate theoretical spectra of all possible peptides without the need for DDA experiments. This paper reviews the research progress of deep learning methods in the prediction of retention time and in related applications in order to provide references for retention time prediction and protein identification. At the same time, the development direction and application trend of retention time prediction methods based on deep learning are discussed.

蛋白质组学对蛋白质进行规模化研究,从蛋白质水平和生命本质层次上研究和发现生命活动的规律和重要生理、病理现象的本质,揭示基因活动的动态表达。基于液相色谱-质谱联用(LC-MS/MS)的“鸟枪法”策略是蛋白质组学研究中应用最广泛的工具^[1]^。在该策略中,蛋白质首先酶解成肽段,利用液相色谱等分离方法将复杂的多肽混合物按照特定性质进行有效的分离后,肽段经过电喷雾电离离子化后进入质谱仪进行谱图采集。通过谱图和数据库比对搜索解析出谱图对应的肽段信息,然后进行组装还原成蛋白质。因此,将肽段的质谱谱图与数据库中的理论序列进行匹配是肽段(以及蛋白质)鉴定、定量和所有随后的生物学解释的核心^[2]^。除了质谱谱图中所提供的肽段母离子和子离子质荷比之外,“鸟枪法”策略还可提供一些额外的数据用于数据分析,从而获得更为准确和全面的肽段序列解析,最常用的是肽段的色谱保留时间(RT)^[3]^。

在蛋白质组学分析中,肽段的色谱保留时间是指在一定的色谱梯度条件下肽段从色谱柱洗脱所需的时间,作为肽段的特性之一与肽段的分子结构、极性和疏水性密切相关。保留时间是独立于质谱分析结果的肽段特征信息,特定肽段的保留时间可以根据肽段的信息(如肽段序列)进行预测,得到的预测保留时间可作为质谱检测的补充辅助进行肽段鉴定^[4]^,以提高肽段鉴定的可信度。保留时间预测在质谱选择性反应监测(SRM)^[5]^、数据依赖性采集方法(DDA)和非数据依赖性采集方法(DIA)^[6]^等流程中均有重要的应用。预测的保留时间通常与相应的质谱数据相结合,用于DDA采集结果的缺失值填充或构建模拟谱图库用于DIA采集结果的搜库^[7]^。本文结合我们课题组多年来在蛋白质组学领域的研究工作,特别是使用预测保留时间辅助一级质谱鉴定的工作,主要综述了基于深度学习的保留时间预测方法的进展及应用。

## 1 传统保留时间预测

传统的保留时间预测采用定量结构保留关系(quantitative structure retention relationship, QSRR)模型,基于肽段的理化性质在特定的色谱条件下对保留时间进行预测^[8]^。这种方法需要对大量标准肽段的保留时间进行测试,建立肽段的保留时间与计算得到的理化性质间关系的模型。保留因子(retention coefficient, Rc)是评价单个氨基酸对保留时间的贡献的参数,一个肽段上所有氨基酸的保留因子之和可以用来估计保留时间。此外还要考虑到肽段长度、电荷数以及螺旋性等因素对保留时间的影响^[9]^。目前应用较多的传统保留时间预测模型有SSRCalc^[10]^, Elude^[11]^和GPTime^[12]^等。这些方法在多个数据集上进行保留时间预测的决定系数(coefficient of determination, *R*^2^)值均小于0.965,预测精度还有提升的空间^[13]^。目前对肽段的理化性质以及肽段与色谱固定相之间复杂的相互作用还没有充分的理解,导致对肽段的保留时间预测结果不够理想^[14]^。而且保留时间预测模型都是在特定的色谱条件下进行训练得到的,如何将模型应用到其他的色谱系统也是一个关键的问题。

## 2 基于深度学习的保留时间预测方法

### 2.1 深度学习

深度神经网络,包括卷积神经网络(CNN)和递归神经网络(RNN)等^[15]^,可以自动学习对象的内在性质,发现大型数据集中的复杂结构。深度学习的特点是叠加多个隐藏层的神经网络,在不需要人为设计特征的情况下提取原始数据。深度学习通过由多个处理层组成的计算模型来学习具有多个抽象级别的数据。这些方法极大地提高了语音识别、视觉对象识别、对象检测和许多其他领域的技术水平。深度神经网络在利用其多层神经元发现数据的复杂结构时非常有效和灵活,使用反向传播算法优化计算层与层之间关系的内部参数,从而发现大数据集中的复杂结构。深度学习也被用于分析LC-MS数据。在蛋白质组学中,深度学习方法已经被用于进行二级质谱谱图预测^[16]^、多肽从头测序^[17]^等流程。

### 2.2 保留时间预测

基于深度学习的保留时间预测方法通常是把肽段的氨基酸序列信息输入到神经网络的隐藏层中,经过各个层之间的复合函数的计算,最终输出预测的保留时间值。通过使用大量的数据对神经网络进行训练,函数参数通过动态路径选择等方法不断优化,使得预测的结果更加准确。

Ma等^[18]^发展了DeepRT方法,使用了8个数据集进行训练、验证和测试,涵盖了不同的物种、肽段修饰状态和液相色谱条件。使用嵌入(embedding)编码的方法,将一个肽段上的每个氨基酸都编码成20维的向量,这个向量能够反映这个氨基酸及其修饰信息,这些向量堆叠形成的矩阵则反映了整个肽段的信息。CNN能够非常有效地检测肽段上氨基酸间的相互作用^[19]^,因此在DeepRT胶囊神经网络(CapsNet)中先通过两层的卷积层处理肽段序列,然后再使用后面的胶囊层计算保留时间。由于色谱条件存在差异,DeepRT无法直接用于新的数据集的预测。深度学习算法可以通过迁移学习的策略,使用小数据集中有限的信息对已经用大量数据预训练过的模型进行校正^[20]^。DeepRT也使用这种方法,先使用其他液相色谱条件下的大量数据进行训练,再使用新的液相色谱条件下的少量数据进行微调校正。在反相液相色谱(RPLC)条件下使用3个数据集进行测试,DeepRT得到的预测值与真实值的*R*^2^达到了0.987、0.970和0.994,比其他保留时间预测软件ELUDE和GPTime的保留时间预测更精确,在强阳离子交换色谱(SCX)和亲水相互作用液相色谱(HILIC)的条件下*R*^2^最高也达到了0.996和0.993。Ma等^[18]^又使用一个包含140000条肽段的大数据集进行训练,得到了改进的DeepRT,称为DeepRT(+),然后使用迁移学习的策略对另外两个数据集进行预测。使用这两个数据集训练得到的DeepRT的预测结果的*R*^2^分别为0.987和0.970, DeepRT(+)迁移学习预测结果的*R*^2^提高到了0.993和0.980。

提高深度学习算法预测的准确性需要使用大量的数据集进行训练。ProteomeTools project提供了一个非常大的合成肽段的液相色谱-质谱联用分析数据库,旨在为人类全部蛋白质和重要的翻译后修饰提供基于合成肽段的高质量质谱数据参考^[21,22]^。Gessulat等^[23]^利用ProteomeTools的数据训练了一个能够精确预测保留时间和离子强度的深度学习算法Prosit。算法通过输入肽段序列、电荷以及标准碰撞能可以输出预测的离子强度和保留时间。其中离子强度预测需要上述3种信息,而保留时间预测只需要肽段序列信息。经过训练,用Proist预测保留时间指数(iRT),预测值与真实值间的相关系数(*R*)值达到了1.00, 95%的置信区间为4.25iRT单位,对应于1 h的LC-MS中的85 s。作为对比,用SSRCalc对同样的数据进行了保留时间预测,结果为*R*=0.96, 95%的置信区间为20.4iRT单位。使用上述模型分别对胰蛋白酶切(tryptic)和糜蛋白酶切(chymotryptic)的肽段进行预测,预测值和观察值间的*R*值分别为0.89和0.91。接着使用迁移学习的方法对模型进行校正,校正后的*R*值分别为0.95和0.98。值得注意的是,上述校正只使用了胰蛋白酶切的数据进行校正,同样也提高了非胰蛋白酶切肽段的预测准确度,预测的iRT也与实验得到的非常一致。这表明Prosit学习了肽段保留时间的一般决定因素,并在各种蛋白酶切条件下推广。这也同样适用于不同的液相色谱环境,当在特定的色谱环境中进行预测时,只需要用部分当前色谱环境下的数据进行迁移学习即可得到精确的预测结果,而不需要使用大量的数据对Prosit进行彻底重新训练。

Guan等^[24]^采用共同的核心架构,双向长短期记忆网络(bidirectional long-short term memory, BiLSTM)建立了3种深度学习预测模型,分别预测了LC-MS/MS中的3种性质:iRT、MS1电荷状态分布以及高能碰撞解离(HCD)碎裂模式下的子离子强度。其中,用来训练iRT预测模型的数据来源于Bruderer等^[25]^的DIA数据,错误发现率(FDR)为1%。经过过滤,共得到了125793条肽段的信息,其中90%用于训练深度学习模型,剩下的10%用于模型的测试。文中提出了一些可能来自于数据集的错误:首先,在此数据集中肽段的FDR为1%,因此至少1%的iRT数据是有误的;其次,iRT与RT间的校正函数也可能带来一定的不确定因素;第三,iRT数据是由多个色谱分离条件整合得到的,分离条件之间的不一致也会导致误差。此外,在iRT预测模型中,唯一允许的修饰是蛋氨酸的氧化。Guan等^[24]^还考察了几种不同的深度学习模型,包括常见的卷积神经网络,以及胶囊神经网络。在当前使用的数据集的条件下,BiLSTM神经网络的表现优于其他神经网络。Guan等把他们训练的模型与DeepRT和Prosit对比发现,Guan等的模型比DeepRT精确28%,而95%的置信区间比Prosit宽了两倍。这可能与二者使用的样本不同有关,Prosit的训练数据集是合成肽集,具有较高的丰度,而Guan等的训练数据则来自于复杂的细胞裂解物样本。以上结果说明研究样本的复杂度和梯度长度对iRT的预测有着重要的影响。

通过迁移可以使用少量数据对基于深度学习的保留时间预测模型进行校准,以实现对不同实验环境下肽段保留时间的预测,这对在数据较少的条件下进行保留时间预测提供了一种有效的方法。对于某一实验环境,若实验数据充足,使用大量同一实验环境的数据对深度学习模型进行完全训练可以使预测更加精确。Yang等^[26]^开发了DeepDIA模型,旨在对特定条件下的二级谱图和保留时间进行更加准确的预测。DeepDIA基于CNN和BiLSTM,输入肽段的序列信息,可以预测出各个可能的b/y离子的相对强度和肽段的iRT信息。DeepDIA预测的iRT与实验得到的iRT间的*R*值大于0.99。当训练数据和测试数据来自于同一实验条件下时,预测的iRT与实验得到的iRT间的四分位范围小于3。另外两次训练数据和测试数据来自于不同实验条件下,二者间的四分位差分别为3.35和5.26。为评估DeepDIA的保留时间预测效果,Yang等^[26]^对DeepDIA、Prosit以及SSRCalc进行了比较。在训练用的数据与测试用的数据来源于不同实验条件的情况下,DeepDIA与Prosit的结果接近,优于SSRCalc;在训练用的数据与测试用的数据来源于相同实验条件的情况下,DeepDIA的预测效果要优于Prosit。

通过深度学习和迁移学习技术,Wen等^[27]^开发了基于肽段序列的保留时间预测工具AutoRT。每个肽段通过独热编码(one-hot encoding)成矩阵形式,具体来说每个氨基酸都被表示为除一项外的所有值都是零的二进制向量,这一项被设置为1来表示氨基酸的类别。特别地,被修饰的氨基酸将会以区别于原氨基酸的形式编码,这样在预测时也能体现被修饰氨基酸的影响。使用了一个从PRIDE^[28]^上获得的大型公共数据集PXD006109^[29]^进行训练,利用遗传算法自动搜索最佳架构。以均方误差(MSE)为标准,选出10个最好的神经系统架构模型,整个模型的训练都是基于这10个神经网络模型。这10个模型经过迁移学习的方法微调后就可以对特定实验条件下的保留时间进行预测。AutoRT根据四分位间距(IQR)算法,去除这10个模型预测结果中的异常值,把剩余结果的平均值作为AutoRT模型整体的预测结果。Wen等^[27]^分别把这10个模型与AutoRT模型整体进行比较,在3个数据集下进行测试。结果表明AutoRT模型整体的中值绝对误差(MAE)平均比各单独的模型低25%、28%和18%。为进一步评估AutoRT的表现,Wen等^[27]^把AutoRT与3个基于深度学习的预测模型Prosit、DeepMass和GuanMCP2019以及一个传统的基于机器学习的工具GPTime在3个大型公共数据集上进行比较,AutoRT的中值绝对误差全部低于其他模型,且4个基于深度学习的模型的中值绝对误差都低于GPTime。

大部分基于深度学习的保留时间预测模型在对输入的肽段信息进行编码时,都是将氨基酸及其位置转化为氨基酸独热编码。然而使用独热编码限制了模型在一些情况下的应用,例如对蛋白质修饰及位点的研究^[30,31]^。独热编码方法在对被修饰的氨基酸进行编码时,每一个潜在的修饰都需要用一个二元特征来表示,而潜在修饰数量众多,使得这种方法实现非常困难。Bouwmeester等^[32]^通过在原子组成的水平上对肽段和修饰进行编码,建立了DeepLC,实现了对修饰肽段的保留时间的精确预测,即使某种修饰在训练数据中没有出现,也能对其进行预测。DeepLC对肽段信息的编码分为4个独立的路径:氨基酸组成、双氨基酸组成、独热编码和全局特征。氨基酸组成路径中,肽段的信息被编码成60×6的矩阵,其中60代表60个氨基酸(不足60个氨基酸的肽段用“X”补足), 6是氨基酸所含6种原子(C、H、N、O、P、S)的个数,被修饰氨基酸的修饰部分的原子数也计入在内,这使模型可以对训练数据中不存在的修饰进行预测。双氨基酸组成路径是将肽段上的氨基酸两两分为一组,互不重叠,矩阵大小为30×6,意义和氨基酸组成路径相同。独热编码路径仅编码了氨基酸非修饰的部分,用来捕捉分子整体的信息,比如区分异构体异亮氨酸和亮氨酸。全局特征路径包括了肽段长度和包含的各原子数目的信息。DeepLC将上述信息整合计算后输出预测的肽段保留时间。经过验证,在对非修饰肽的保留时间预测上,DeepLC与目前最先进的模型DeepRT^[18]^、Prosit^[23]^以及Guan等^[24]^的模型表现相近。经过更大的数据集训练后DeepLC的表现进一步提高,通过迁移学习能够对小的数据集提供准确的预测。更重要的是,DeepLC能准确地预测被修饰肽段的保留时间,对没有在训练的数据集里出现的修饰也能准确预测。但是对于复杂的修饰,如糖基化修饰,保留时间的预测结果还不够准确。如何进一步提高预测修饰肽段的准确性是研究的重要方向。

## 3 基于深度学习的保留时间预测方法的应用

保留时间为基于液相色谱-质谱联用的肽段鉴定提供了一个额外维度的信息^[14]^,可以应用到蛋白质组学分析工作流程的多种任务中。本课题组在校正保留时间的基础上,进行一级质谱水平上的精确质量数匹配和质谱峰提取,显著降低了完整*O*-GalNAc糖肽鉴定缺失的问题,同时插补得到定量数值^[33]^。通过对肽段的保留时间预测,可以提高质谱鉴定的准确性^[34, 35]^,也有助于设计更加高效的实验^[36]^,以及鉴定嵌合碎片谱图^[37]^。随着蛋白质组学其他技术的发展,保留时间的预测也有了其他的应用。近年来,许多研究将保留时间预测模型与碎片峰离子强度预测模型相结合,生成了全面的模拟数据库,用于进行DIA的搜库,有效地替代和超越了基于DDA的经验数据的谱图库^[38]^。基于深度学习的保留时间预测方法也被应用于提高质谱鉴定的准确性和可靠性、生成全面的模拟数据库等方面。接下来,本文将对前文介绍的基于深度学习的保留时间预测方法的应用进行综述。

### 3.1 预测DIA谱图库

DIA是一种强大的质谱数据采集技术,可用于深度全面的蛋白质质谱分析^[6,39]^。通过DIA,质谱仪可以将所有的信号按照固定的质荷比和保留时间划分为许多区域,然后对每块区域里的所有一级信号全部一次性进行二级采集,从而消除了DDA模式的随机性带来的数据丢失集。DIA通常使用由DDA实验得到的数据建立谱图库进行肽段鉴定^[40]^,构筑这些DIA谱图库需要花费大量的时间、样本和精力,而且通常不能跨实验室或仪器平台使用^[25]^。此外,这种谱图库构建的方法也把DIA定性和定量的对象限定在了由DDA鉴定出的肽段上,反而限制了DIA方法无损检测的固有优势。因此,建立包含预测的保留时间和碎片离子信息的谱图库具有重要意义。有许多传统模型被用来预测保留时间和碎片离子信息^[41,42]^,但仍局限在特定的实验室和仪器平台上。随着深度学习在蛋白质组学的应用,基于深度学习的保留时间预测模型和碎片离子预测模型被结合在一起,用于构建模拟库进行DIA搜库。Gessulat等^[23]^为了测试开发的Prosit建立模拟库的效果,分别对4个来自于不同物种的公共谱图库中的肽段进行模拟建库,然后与这4个谱图库进行比较。Prosit建立的模拟库与4个实测谱图库非常相近,谱角顶点(apex of spectral angle)达到了0.9, *R*值大于0.95。然后Gessulat等^[23]^又使用在特定仪器平台条件下得到的DIA数据分别检索Prosit建立的模拟谱图库与在该平台获得的高质量实测谱图库,分别得到了6739和6919种蛋白质。Prosit模拟谱图库的效果比高质量的实测谱图库略差,但可以取代一些低质量或是高信噪比的谱图库,能够提高近20%的肽段鉴定数量。

Tiwary等^[43]^开发的深度学习方法DeepMass: Drip结合了母离子的保留时间预测与二级质谱谱图预测,可以生成模拟谱图库。为了测试DeepMass: Drip的效果,Tiwary等^[43]^对DDA库中的7441条肽段的碎片离子强度和保留时间进行预测并建库,然后使用Spectronaut进行DIA搜索。得到的平均定量肽段数目为4957条,比用DDA数据建库进行DIA搜索得到的肽段数目少291条(5.5%)。然而,模拟库搜索少鉴定到的这些肽段在搜索DDA数据库时Spectronaut的打分也较低,其中118条(41%)的最小FDR阈值大于10^-3^。

使用预测的模拟谱图库进行DIA搜索存在两个不利因素:首先,由于模拟库包括了蛋白质中所有可能存在的肽段,与只包含检测到的肽段的实测谱图库相比控制假阳性率需要更高的阈值;其次,虽然深度学习的方法能够得到比其他传统方法更高质量的预测谱图库,这些预测的准确性仍然要低于在该试验条件下由实验得到的数据。Searle等^[44]^基于色谱库^[45]^的方法,对预测的谱图库进行修正,得到了更高质量的谱图库用于DIA搜库。首先使用Prosit对蛋白质序列数据库中所有可能的胰蛋白酶解肽段的碎片离子和保留时间进行预测,建立预测的谱图库。然后按照色谱库的方法,使用该预测谱图库对6次DIA数据进行搜库,用得到的肽段鉴定结果建立了一个特定实验条件下的修正的谱图库。这个新的谱图库只包含了这6次DIA搜库鉴定出的肽段碎片离子信息和保留时间,在该实验条件下DIA实验得到的数据比原本预测的数据更加准确。Searle等^[44]^将这个修正的数据库用于单次DIA数据的搜库。使用酵母样本进行单次DIA实验,使用该修正的库鉴定到的肽段数量比使用DDA库鉴定到的肽段数量提高了31%。

血浆蛋白质组学为一系列疾病的蛋白质生物标志物的发现带来了巨大希望^[46,47]^,然而血浆中蛋白质丰度极大的动态范围(超过12个数量级)阻碍了血浆蛋白质组学的发展。Yang等^[26]^使用其开发的DeepDIA建立了血浆蛋白的模拟谱图库,使用该谱图库进行DIA搜库,在未经高丰度蛋白质去除的条件下,平均每次可以鉴定到超过400种蛋白质,两倍于最先进的DDA数据库鉴定到的蛋白质数目。通过在样品中掺入稳定同位素标记的参比肽段的评估方法,发现使用模拟谱图库鉴定的错误率与使用DDA建立的谱图库相近。

### 3.2 质量控制

人类肿瘤通常有多个体细胞突变,它们的转译可能产生新抗原,这些新抗原是基于t细胞的癌症免疫治疗的理想目标,因为它们是免疫系统的外来物^[48]^。一些寻找和发现新抗原的方法依赖于蛋白质组学中对变异肽高敏感度和可靠性的鉴定。在蛋白质组学分析中,通常由反库等方法估测和控制FDR来进行质量控制^[49]^,然而普通的FDR控制方法没有对变异肽和普通肽进行区分,由于变异肽在实际实验中发现的可能性较低,这种全局FDR方法对变异肽的FDR会偏低,容易出现假阳性^[50]^。为解决这一问题,可以使用另外两种FDR控制方法:单独FDR方法(separate FDR method)分别计算已知肽段的FDR和变异肽段的FDR^[51]^;两级FDR方法先基于参照蛋白质数据库进行搜库,去掉鉴定到的高可信度的谱图,再用剩下的谱图基于变异蛋白质数据库搜库,并计算变异肽的FDR^[52]^。PepQuery等工具可以对通过FDR的变异肽进行校检,有助于降低假阳性率^[53]^。Wen等^[27]^通过基于深度学习的保留时间预测工具对各种质量控制方法进行评估,其原理为肽段的保留时间可以通过肽段序列进行预测,是肽段的固有特征,独立于FDR,预测的保留时间与观察到的保留时间的差异可以作为一个有效的、无偏的指标来评价不同的肽段鉴定方法中肽段和谱图匹配(PSM)的质量,差异越大,则PSM质量越低。Wen等^[27]^以上述3种FDR控制方法以及是否使用PepQuery进行后续质量控制作为变量,对287个肿瘤样本进行实验,通过预测保留时间和实际保留时间的差异来评价各种方法,证明使用全局FDR方法并使用PepQuery进行后续校验的灵敏度最高,并且也证明了基于保留时间的校正为降低假阳性提供了一个额外的过滤方法,可以提高发现变异肽的可靠性。

## 4 总结与展望

基于深度学习的保留时间预测方法具有可通过多层神经网络自动从复杂的数据中学习、准确度高、可应用于不同的实验环境等优点,而且与其他大型深度学习方法相比,使用单独的保留时间预测方法对硬件的要求并不高,这也有利于保留时间预测方法的应用。目前对于保留时间预测方法的研究主要有以下几个方向:一,优化模型,以及使用数据量更大、准确度更高的数据集进行训练,进一步提高保留时间预测的准确度;二,提高模型在不同实验环境下的适用性,目前的方法是预测iRT和通过迁移学习在新环境下对模型进行校正;三,优化编码方法,提高对修饰肽段保留时间预测的准确性。大部分模型对修饰肽段的预测能力非常有限,需要在训练模型和进行预测时把不同修饰的修饰位点的氨基酸进行特定编码,与未修饰的氨基酸进行区分,这种方法难以适用于修饰种类和位点较多的情况,而且由于训练用的数据集中的修饰不一定包含需要的修饰,在使用时通常需要重新训练模型。DeepLC模型对各种修饰在原子水平上进行编码,能够反映修饰的原子组成对保留时间的影响,解决了前面的两个问题,但难以反映修饰的结构对保留时间的影响。当修饰较大和较复杂时,如糖基化修饰,修饰的结构对保留时间有较大的影响,所以如何反映修饰结构的影响也是一个重要的研究方向。

目前对保留时间预测的应用大多集中在与谱图预测相结合,建立模拟的谱图库用以DIA等方法的搜库,也用于质谱方法的评估和质量控制等方面。随着保留时间预测的准确度和适用性的进一步提高,保留时间作为液相色谱-质谱联用结果中的一个重要信息维度,将会在蛋白质组研究中发挥更加重要的作用。
